# Longitudinal health-related quality of life profiles in esophageal cancer: insights from a nationwide prospective observational cohort study

**DOI:** 10.1007/s11764-026-01968-9

**Published:** 2026-01-19

**Authors:** Esther Deuning-Smit, Julien A. M. Vos, Ehsan Motazedi, Ellen Engelhardt, Shosha H. M. Peersman, Cecile V. Baartman, Annemarie M. J. Braamse, Monique Borremans-Simons, Merlijn Hutteman, Ewout A. Kouwenhoven, Sjoerd M. Lagarde, Grard A. P. Nieuwenhuijzen, Jelle P. Ruurda, Marije Slingerland, Fabiola Müller, Hanneke van Laarhoven, Pieter Coenen

**Affiliations:** 1 Department of IQ Health, Radboud University Medical Center, Nijmegen, the Netherlands; 2 Department of General Practice, Amsterdam, UMC Location University of Amsterdam, Amsterdam, the Netherlands; 3 Cancer Center Amsterdam, Cancer Treatment and Quality of Life, Amsterdam, the Netherlands; 4 Department of Public and Occupational Health, Amsterdam , UMC Location Vrije Universiteit, Amsterdam, the Netherlands; 5 Societal Participation and Health, Amsterdam Public Health Research Institute, Amsterdam, the Netherlands; 6 Division of Psychosocial Research and Epidemiology, Netherlands Cancer Institute, Amsterdam, the Netherlands; 7 Princess Máxima Center for Pediatric Oncology, Utrecht, the Netherlands; 8 Department of Medical Oncology, Amsterdam, UMC Location University of Amsterdam, Amsterdam, the Netherlands; 9 Department of Medical Psychology, Amsterdam, UMC Location University of Amsterdam, Amsterdam, the Netherlands; 10 Department of Surgery, Elisabeth-TweeSteden Hospital, Tilburg, the Netherlands; 11 Department of Surgery, Radboud University Medical Center, Nijmegen, the Netherlands; 12 Department of Surgery, ZGT Hospital, Almelo, the Netherlands; 13 Department of Surgery, Erasmus Medical Center, Rotterdam, the Netherlands; 14 Department of Surgery, Catharina Hospital, Eindhoven, the Netherlands; 15 Department of Surgery, University Medical Center Utrecht, Utrecht University, Utrecht, the Netherlands; 16 Department of Medical Oncology, Leiden University Medical Center, Leiden, the Netherlands

**Keywords:** Esophageal neoplasms, Cancer, Quality of life, Chemo-radiotherapy, Latent class analysis, Longitudinal studies

## Abstract

**Purpose:**

Identifying patients with similar health-related quality of life (HRQoL) recovery patterns can guide more effective disease management. This study aimed to identify cross-sectional and longitudinal HRQoL profiles in esophageal cancer patients, and to examine associations with demographic and clinical characteristics.

**Methods:**

Longitudinal data from the Prospective Observational Cohort Study of Esophageal-gastric cancer Patients (POCOP) were analyzed. Patients treated with chemoradiation (CRT), with/without surgery, were followed for 12 months post-treatment. HRQoL was measured pre-treatment, at 6- and 12-months using validated questionnaires (i.e., EORTC QLQ-C30, QLQ-OG25, and HADS). Latent Profile and Latent Transition analyses were used to identify cross-sectional and longitudinal profiles, respectively. Regression analyses explored their associations with demographic and clinical characteristics.

**Results:**

Of the 605 patients (mean age: 66.6 (SD: 8.0) years), most were male (82%) and had undergone surgery (73%). At each time point, two cross-sectional HRQoL profiles were identified: one with relatively high HRQoL (78–84%) and low HRQoL (16–22%). Both profiles showed significant impairments compared to population norms. Longitudinal analyses revealed five distinct HRQoL profiles: stable-high (65%), stable-low (5%), improving (10%), deteriorating (14%), and fluctuating (6%). Poorer HRQoL was associated with female sex and lower body mass index. Fluctuating HRQoL with surgery.

**Conclusions:**

In our cohort, most esophageal cancer patients experienced relatively high or improving HRQoL, but some faced deteriorating or persistent impairments. Patients’ sex, BMI, and surgery status may help identify those needing extra support.

**Implications for Cancer Survivors:**

Recognizing different HRQoL profiles can help manage recovery expectations, and guide more personalized and timely care after treatment.

**Supplementary Information:**

The online version contains supplementary material available at https://doi.org/10.1007/s11764-026-01968-9.

## Background

Esophageal cancer ranks as the eighth most common type of cancer worldwide, with the sixth leading cause of cancer deaths, characterized by its poor prognosis [1, 2]. Surgical resection with or without neoadjuvant chemoradiation (CRT) remains the cornerstone of curative treatment for esophageal cancer. CRT alone is sometimes used as an alternative, particularly for patients with poor functional status, irresectable tumors, or those unwilling to undergo surgery [3]. After treatment, esophageal cancer patients often experience various physical and psychosocial symptoms [4, 5]. These include common cancer-related symptoms, such as fatigue and anxiety, as well as symptoms more specific to esophageal cancer and its treatment, such as difficulties with eating, drinking, and regurgitation. The symptom burden among esophageal cancer patients is significantly higher than in the general population and may persist for up to 10 years post-surgery [6–8].

Given the high prevalence and severity of symptoms, previous studies have attempted to identify symptom clusters in esophageal cancer patients [9–11]. Symptom clusters are defined as the co-occurrence of two or more symptoms that are stable, independent of other clusters, and may share underlying mechanisms and/or outcomes [12, 13]. While evidence supports the existence of symptom clusters in esophageal cancer [9–11], inconsistencies between studies make it difficult to apply these findings clinically. Since symptoms often fluctuate or diminish over time, it could be more informative to examine patients’ health-related quality of life (HRQoL). Health-related quality of life (HRQoL) involves more than just symptoms, incorporating various physical, emotional/mental, and social aspects. HRQoL is a well-established endpoint in cancer research and shows prognostic value for survival [14–16]. Patients with similar HRQoL patterns can be categorized into profiles. Identifying these profiles may offer a more robust understanding of how esophageal cancer impacts patients’ lives. Furthermore, HRQoL outcomes are relevant to surgical decision making and help guide personalized treatment strategies [17]. Therefore, identifying patient subgroups who are at risk of poor HRQoL may enable more effective and efficient disease management.

In this study, we used longitudinal data from the Prospective Observational Cohort Study of Esophageal-gastric cancer Patients (POCOP) to identify cross-sectional and longitudinal HRQoL profiles in esophageal cancer patients, and to examine associations with demographic and clinical characteristics. 

## Methods

We used longitudinal data from POCOP, a nationwide population-based, observational cohort study with patient-reported data in the Netherlands [18]. Additional clinical variables for the study population were obtained through linkage with the Netherlands Cancer Registry (NCR), a nationwide register providing cancer diagnosis and treatment-related variables for all cancer patients in the Netherlands. STROBE guidelines were used for the reporting of the study [19].

### Patients and procedures

Details about the recruitment procedure and design of the POCOP study are reported in detail elsewhere [18]. In brief, patients were eligible for participation in the POCOP study if they were diagnosed with esophageal or gastric cancer from December 2015 onwards. Recruitment for the study takes place at 62 medical centers in the Netherlands. All patients provide written informed consent for study participation and allow their data to be linked with the NCR.

In our analyses, we included patients who: (1) were diagnosed with potentially curable esophageal cancer (cT1-4A N0-3 M0) between December 2015 and December 2019, (2) received CRT with or without surgery, (3) provided consent to have their data linked to NCR and (4) completed at least 1 POCOP questionnaire. We used the POCOP assessments at baseline (i.e., pre-treatment), 6 months, and 12 months after the completion of CRT. Because there was variability in the time of recruitment and questionnaire completion, the actual completion date of measurements was not always in line with the intended time point according to POCOP (baseline, 6 and 12 months). During data cleaning, we therefore excluded measurements that were completed more than 6 months before or after the intended time point to prevent overlap between time points.

### Measurements

Table 1 provides an overview of the sociodemographic and clinical variables as well as the outcome measures. Outcomes included validated items regarding health-related quality of life (HRQoL). These items were selected prior to analyses by the research team based on their clinical relevance for esophageal cancer. Normative data from the general population were used as reference values [20–22].

**Table 1:** Overview of the various measurements used in the POCOP study

**HRQoL**	**EORTC QLQ-C30** [23] The EORTC QLQ-C30 is a core questionnaire consisting of 30 items that assess general aspects of cancer patient's HRQoL. The responses are scored on a 4-point Likert scale and grouped in 5 functioning scales and 3 symptom scales. Total scores for each scale can be calculated and range from 0 to 100. Scores were transformed so that higher scores denote poorer functioning or more severe symptoms. The Global Health/QoL scale was excluded because it overlaps with most other items and provides limited information for clinical interpretation of the profiles. In this study, we included the following: - Functional scales (reference mean ± SD): Physical (5 items, 13.0 ± 16.7); Role (2 items, 13.3 ± 22.6); Emotional (4 items, 12.7 ± 17.0); Cognitive (2 items, 8.7 ± 13.4); Social (2 items, 7.8 ± 16.8) - Symptom scales (reference mean ± SD): Fatigue (3 items, 20.1 ± 22.8); Nausea and vomiting (2 items, 3.5 ± 9.7); Pain (2 items, 20.7 ± 24.7)
	**EORTC QLQ-OG25** [24] The EORTC QLQ-OG25 consists of 25 items that specifically assess symptoms related to esophageal or gastric cancer and the impact of the disease and its treatment on the patient's social and emotional functioning. The responses are scored on a 4-point Likert scale. Total scale scores can be calculated and range from 0 to 100, with higher scores indicating more severe symptoms or greater impact on quality of life. In this study, we included the following: - Scales (reference mean ± SD): Dysphagia (3 items, 0.6 ± 5.1); Eating restrictions (4 items, 2.5 ± 9.0); Reflux (2 items, 7.0 ± 15.8); Odynophagia (2 items, 1.6 ± 8.6); Pain and discomfort (2 items, 7.8 ± 15.7); Anxiety (2 items, no reference value) - Single items (reference mean ± SD): Eating in front of others (1.4 ± 8.3); Dry mouth (11.6 ± 22.7); Trouble with taste (2.2 ± 11.1); Body image (no reference value); Trouble swallowing saliva (1.0 ± 7.0); Choked when swallowing (3.2 ± 12.5); Trouble with coughing (13.2 ± 23.2); Trouble talking (1.7 ± 9.0); Weight loss (1.3 ± 8.5)
	**Hospital Anxiety and Depression Scale (HADS)** [25] The HADS is a questionnaire used to screen for anxiety and depression in patients in a hospital or primary care setting. The HADS consists of 14 items scored on a 4-point Likert scale and is divided into an Anxiety subscale (7 items, reference mean ± SD: 4.9 ± 3.4) and a Depression subscale (7 items, 5.5 ± 3.9). The total score ranges from 0 to 42, with higher scores indicating greater levels of anxiety or depression
**Sociodemographic variables (self-reported at baseline questionnaire)**	- Age (treated as a continuous variable) - BMI (in kg/m^2^; treated as a continuous variable) - Sex (male vs. female) - Education level (low (i.e., elementary school or lower) vs. intermediate (i.e., secondary and post-secondary vocational education) vs. high (i.e., higher vocational education or university)) - Marital status (married or living together vs. single)
**Clinical variables (obtained via linkage with the NCR)**	- Tumor stage (stage I/II vs. stage III) - Topography (location of the tumor; upper esophagus vs. middle/lower esophagus vs. other) - Surgery (no surgery vs. surgery) - No. surgical complications (treated as a continuous variable)

### Statistics

Descriptive statistics were computed to describe the sample characteristics. To answer our research questions, we used a three-step approach. First, we identified patients’ cross-sectional HRQoL profiles at each time point separately (baseline, 6 months, and 12 months) using Latent Profile Analysis (LPA). Next, we tracked changes in HRQoL profiles over time with Latent Transition Analysis (LTA) to describe how patients’ HRQoL evolved across the three time points. Both LPA and LTA analyses are unsupervised learning methods, meaning they identify patterns in the data without using pre-defined labels. Three longitudinal profiles were merged post hoc because they had similar HRQoL recovery patterns. This decision was made to improve the clinical interpretation of the profiles and to address small sample sizes, as seen in other studies [26]. Analyses were performed using the standardized HRQoL measures described in Table 1 as the profile variables. Finally, we examined how demographic and clinical factors (e.g., age, gender, and treatment type) influenced patients’ longitudinal HRQoL profiles using inverse probability weighted multinomial regression analyses*.* The statistical methods are described in full detail in the Supplementary Information (SI p4). The descriptive analyses were conducted in SPSS version 28.0.1.1, the LPA and multinomial regression analyses in R 4.3.1, and LTA in MPlus version 8.9.

## Results

**Fig. 1:**
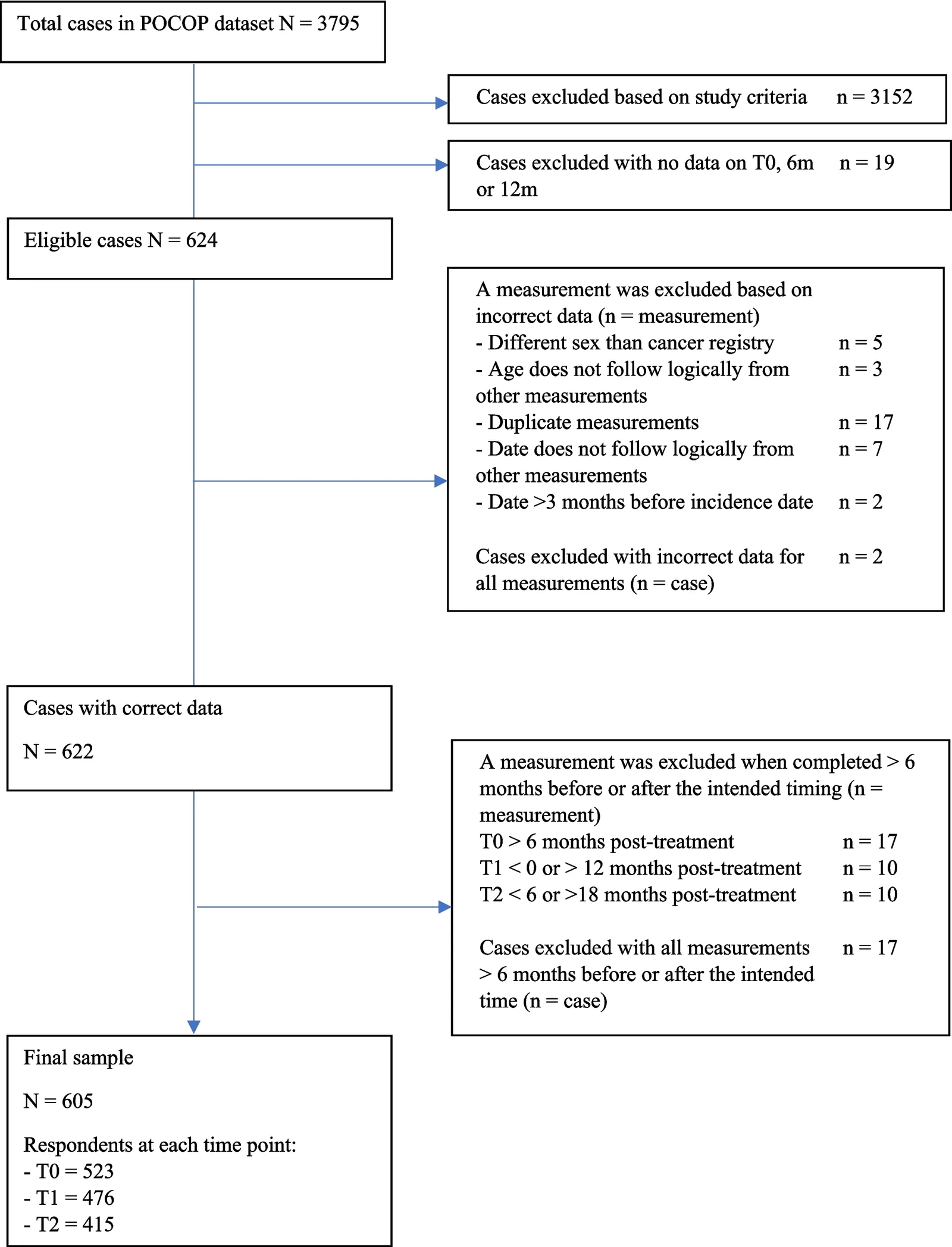
Flowchart of data cleaning

Figure 1 shows the selection of participants. Of the 3,795 participants in the POCOP database, 624 met the eligibility criteria for this study. Nineteen participants were excluded during data cleaning, resulting in a final sample of 605 patients. The mean time to questionnaire completion was 1.3 months (SD: 0.8) at T0, 4.7 months (SD: 1.1) at T1, and 10.8 months (SD: 1.1) at T2. The number of respondents at each timepoint was 523, 476, and 415, respectively. Baseline characteristics of the included patients are shown in Table 2. The sample had a mean age of 66.6 years (SD: 8.0). The majority was male (82%), had a low or intermediate education level (77% combined), and had undergone surgery (73%).

**Table 2:** Baseline (T0) characteristics of the included patients (*n* = 605)

		***n* (%)**	**Mean (SD), [range]**
Sex	Males	497 (82%)	
	Female	108 (18%)	
Education level (*n* = 520)	Low	230 (44%)	
	Intermediate	172 (33%)	
	High	118 (23%)	
Marital status (*n* = 522)	Married/living together	419 (80%)	
	Single	103 (20%)	
Tumor stadium (clinical)	I/II	337 (56%)	
	III	268 (44%)	
Topography	Upper/middle	76 (13%)	
	Lower	465 (77%)	
	Other	64 (11%)	
Surgery	No	164 (27%)	
	Yes	441 (73%)	
Age			66.6 (8.0), [39 85]
BMI (*n* = 494)			26.4 (4,3), [14.6 39.6]
No. surgical complications (*n* = 344)			1.6 (2.2), [0 9]

### Identification of HRQoL profiles

#### Cross-sectional analyses

At baseline and at 6 months, the optimal cross-sectional HRQoL model included two latent profiles that could be characterized as relatively high HRQoL (78–84%) and low HRQoL (16–22%). Profile frequencies are shown in in Table 3. At 12 months, the optimal model included five latent profiles. However, the second best model at this time point included two latent profiles, with a goodness-of-fit only slightly inferior to the so-called best model (shown in SI Table 1). To simplify interpretation and avoid overfitting in LTA, we selected the two-profile model at 12 months, as was done at baseline and at 6 months. The two latent profiles indicated a strong correlation among HRQoL subscales: patients who scored high or low on one subscale (e.g., pain) were likely to score similarly high or low on others (e.g., fatigue, functioning, or nausea). Compared to the general population norms, both the high and low HRQoL profiles showed significant impairments across multiple HRQoL subscales, particularly in the EORTC QLQ-OG25 subscales (SI Table 2). For example, dysphagia scores were 17.9 (SD: 19.6) and 40.6 (SD: 28.0) in the high and low HRQoL profiles, compared to 0.6 (SD: 5.1) in the general population. Similarly, eating scores were 24.2 (SD: 23.1) and 56.4 (SD: 27.4) compared to 2.5 (SD: 9.0). In contrast to the QLQ-OG25 subscales, only the low HRQoL profile showed worse outcomes on the QLQ-C30 subscales. For example, fatigue scores were 17.8 (SD: 16.5) and 52.4 (SD: 20.0), compared to 20.1 (SD: 22.8) in the general population. No reference data were available for the anxiety subscale in the QLQ-OG25, but the low HRQoL profile also had higher anxiety and depression scores on the HADS (8.0 (SD: 4.3) and 7.8 (SD: 3.6), respectively).

**Table 3:** Identified profile frequencies

		***N* (%)**
**Cross-sectional profiles T0 (baseline)**	Low performing profile	95 (16%)
	High performing profile	510 (84%)
**Cross-sectional profiles T1 (6 m)**	Low performing profile	131 (22%)
	High performing profile	474 (78%)
**Cross-sectional profiles T2 (12 m)**	Low performing profile	119 (20%)
	High performing profile	486 (80%)
**Longitudinal profiles (T0-T1-T2)**	low-low-low performing	30 (5%)
	low-low–high performing	7 (1%)
	low–high-low performing	6 (1%)
	low–high-high performing	52 (9%)
	high-low-low performing	62 (10%)
	high-low–high performing	32 (5%)
	high-high-low performing	21 (4%)
	high-high-high performing	395 (65%)
**Longitudinal profiles (combined)**	Stable high performing	395 (65%)
	Stable low performing	30 (5%)
	Improving (i.e., low-low–high, low–high-high)	59 (10%)
	Deteriorating (i.e., high-low-low, high-high-low)	83 (14%)
	Fluctuating (i.e., low–high-low, high-low–high)	38 (6%)

#### Longitudinal analyses

**Fig. 2:**
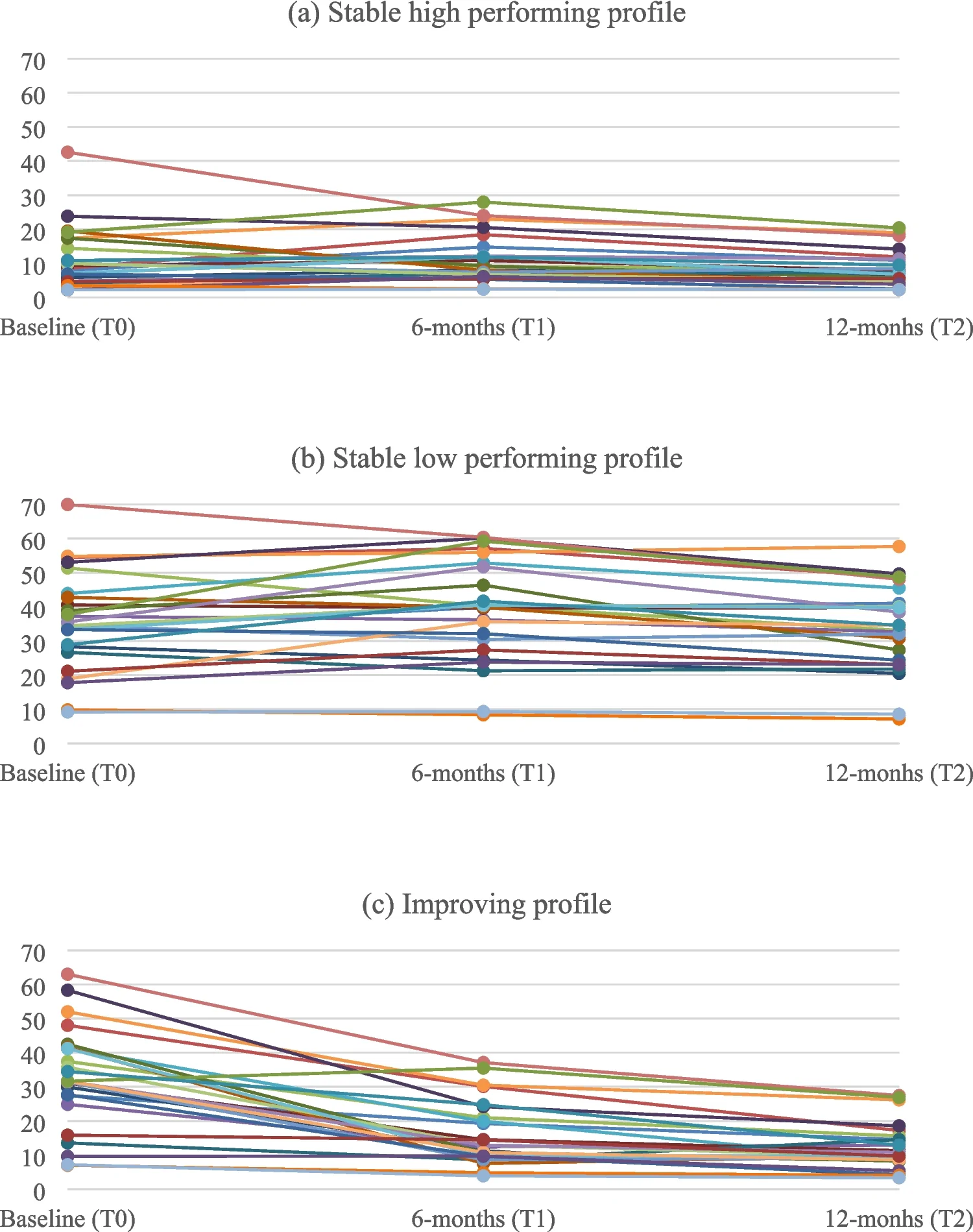

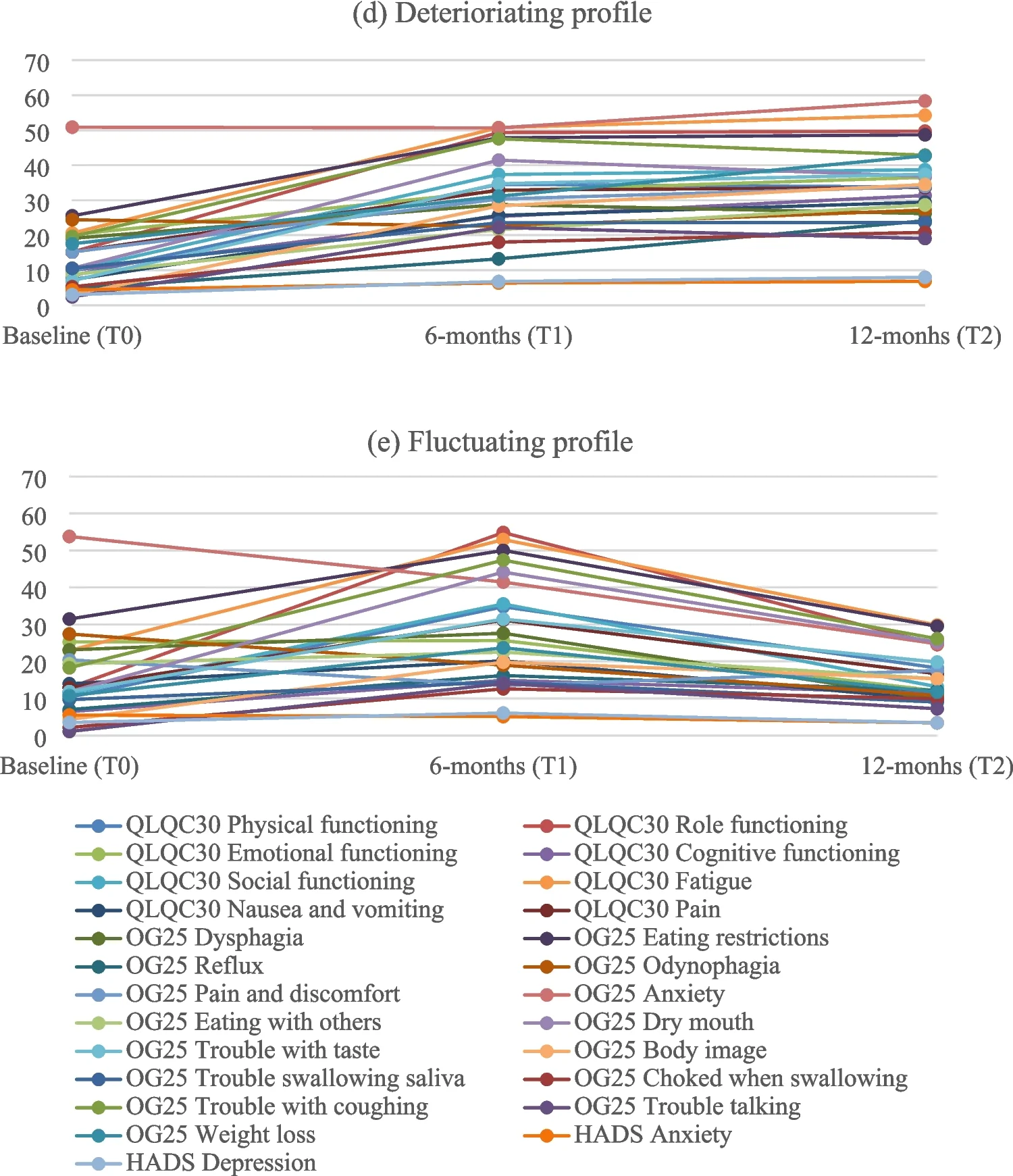
**a–e **Questionnaire scores for each longitudinal health-related quality of life (HRQoL) profile. These figures illustrate the HRQoL patterns across the profiles. Individual scores for each profile can be found in Supplementary Information (p7-8). Interpretation of scores: higher scores denote poorer functioning or experiencing more symptoms

With two profiles at each time point, the LTA included ${2}^{3}=8$ possible longitudinal profiles. Profile membership probabilities were calculated for each time point, conditional on membership at the previous time point. The entropy of the fitted model was 0.823, indicating a good fit. Due to the small number of individuals in some of the eight longitudinal profiles (Table 3), we merged 3 profiles post hoc based on similarities in the direction of the HRQoL changes (e.g., low-low–high and low–high-high were combined into an improving HRQoL profile). This resulted in five longitudinal profiles with the following clinical interpretations: stable-high performing (*n* = 395, 65%), stable-low performing (*n* = 30, 5%), improving (*n* = 59, 10%), deteriorating (*n* = 83, 14%) and fluctuating (*n* = 38, 6%). In the stable-high performing profile, only the anxiety subscale and trouble with coughing item were significantly worse compared to in the general population. After 12 months, no significant impairments were observed in either the stable-high performing or improving profiles. Figure 2 graphically presents the HRQoL values at baseline, 6- and 12 months for each of the five longitudinal profiles. Mean HRQoL scores and norm data are displayed in SI Table 3.

### Association between the identified longitudinal profiles and demographic and clinical characteristics

Sociodemographic and clinical predictors of the longitudinal profiles are shown in SI Table 4 and 5. The stable-high longitudinal profile served as the reference group. Being female, compared to male, was associated with higher odds of stable-low profile membership (OR [95%]: 2.63 [1.18; 5.89]). A one-unit increase in the BMI value was associated with lower odds of membership of the deteriorating profile (OR [95%]: 0.91 [0.85; 0.98]). Finally, having undergone surgery, compared to no surgery, was associated with higher odds of fluctuating profile membership (OR [95%]: 2.68 [1.02; 7.03]).

## Discussion

### Main findings

This study aimed to identify cross-sectional and longitudinal HRQoL profiles among patients with esophageal cancer treated with curative intent, and to examine the associations between these profiles and key demographic and clinical characteristics. Cross-sectional analyses revealed two distinct HRQoL profiles at each of the three time points, i.e., one representing patients with relatively high HRQoL (78–84%) and one with low HRQoL (16–22%). Of note, both profiles showed significant impairments across multiple HRQoL subscales when compared to general population norms, which underscores the substantial impact of esophageal cancer treatment.

Longitudinal analyses revealed five HRQoL profiles, i.e., stable-high performing (65%), stable-low performing (5%), improving (10%), deteriorating (14%), and fluctuating (6%). Although most patients maintained a relatively high HRQoL or improved over time, a subgroup experienced ongoing or increasing challenges, emphasizing the importance of early identification and support for patients at risk of poor HRQoL. Specific sociodemographic factors, such as female sex, lower BMI, and undergoing surgery, were associated with HRQoL recovery.

### Clinical implications

Our results offer valuable insights into the diverse experiences of patients with esophageal cancer following curative treatment. Cross-sectional analyses indicated a strong correlation among aspects of HRQoL: patients who scored either high or low on a certain HRQoL subscale were likely to score similarly on other subscales. This highlights the importance of comprehensive HRQoL assessments, particularly for patients with lower HRQoL, as they are more likely to experience multiple challenges. Longitudinal analyses identified a small but important subgroup of patients with consistently low (5%) or deteriorating HRQoL (14%), emphasizing the need for proactive monitoring. These results are consistent with a recent Swedish cohort study, which found that symptom trajectories with higher severity either persisted or improved over time, whereas milder symptom trajectories remained relatively stable [27].

Clinical guidelines already advocate for a multidisciplinary approach during the follow-up phase, coordinated by the health professional who sees the patient regularly[28]. In this context, the HRQoL profiles clearly support the need for early referrals to supportive care services such as psychological counseling, support groups, and rehabilitation. The identified risk factors can help in recognizing patients at greater risk for long-term HRQoL impairments. Furthermore, HRQoL profiles can help clinicians set more realistic expectations for patients, inform treatment decisions, and support shared decision-making [17]. They can also highlight which HRQoL issues need attention and encourage conversations with patients about their recovery that go beyond just physical symptoms [29]. However, further research is needed to understand how different care approaches influence HRQoL outcomes [29]. An important concept to consider is response shift, which refers to a change in how patients view their own health [30]. This shift doesn’t result from changes in physical health, but from changes in a patients’ internal standards, values, and conceptualization of HRQoL. As a result, HRQoL experiences can change over time, even if their physical health remains stable. To better understand and address these concerns, it is important to engage in open conversations with patients about how they view their health and recovery.

### Strengths and limitations

Prior studies have focused on symptom clusters [9–11], but these clusters do not capture the different aspects of recovery. For example, some patients may maintain high physical functioning scores, but still experience significant social or emotional challenges, whereas others may experience ongoing dysphagia. This heterogeneity in recovery is not adequately modeled when only symptoms are assessed, as the relationships between different scales are lost [31]. Therefore, our study shifts the focus to HRQoL profiles, offering a more comprehensive view of recovery after esophageal cancer and highlighting the need for further research in this area. A key strength of our study is the use of a longitudinal dataset, which includes a broad range of personal and clinical data. We evaluated a large set of general cancer- and cancer-type-specific HRQoL variables, covering physical, emotional/mental, and social domains. HRQoL data were analyzed up to 12 months following treatment, a period during which most of the HRQoL changes are typically observed [8]. The decision to use LPA and LTA allowed us to take into account the probability of individuals transitioning between profiles over time, which provides a more nuanced view of HRQoL recovery.

Some limitations should be noted. First, the NCR data did not provide enough detail to distinguish between patients who received definitive CRT from those who received neoadjuvant CRT without surgery. Since these two patient groups may experience different HRQoL recovery, further research is needed to explore the differences. Second, some longitudinal HRQoL profiles were merged post hoc into broader subtypes. While this approach may overlook some nuances, this decision was based on similarities in HRQoL changes and improved the interpretability of the profiles. The relatively small sample size also limited our ability to perform multivariate analyses. As a result, we may have missed relevant predictors due to limited statistical power and it remains uncertain whether the univariate predictors we identified would hold in multivariate models. Future research should aim to validate these findings and explore the mechanisms underlying HRQoL profiles (e.g., psychological factors such as coping strategies). Third, we did not have data on patients’ HRQoL before cancer diagnosis, which would offer valuable context for interpreting the HRQoL profiles. We also had a relatively broad timespan of 6 months between the follow-up questionnaires, which means we may have missed HRQoL changes that happened within that period. Nevertheless, this choice was justified as sufficient time is needed between measurements to identify meaningful patterns and adding additional time points would increase model complexity without adding much extra information. Fourth, due to the absence of Dutch reference values for the EORTC QLQ-OG25 and HADS, we used the Swedish and German reference values [21, 22]. While we do not believe this significantly affected our results, further research is necessary to establish Dutch population norms. Finally, due to the design of the POCOP study, potential selection bias may have occurred, as we did not have data on the total number of eligible patients or reasons for declining participation.

## Conclusions

This study identified distinct cross-sectional and longitudinal HRQoL profiles in esophageal cancer patients treated with curative intent. While most patients were in a stable-high performing HRQoL profile, a considerable subgroup experienced consistently low or deteriorating HRQoL. Early identification of these patients is important, as it allows clinicians to provide timely and targeted intervention to better support them. Further research is needed to validate our results in larger cohorts and to further explore what drives the HRQoL experiences.

## Supplementary Information

Below is the link to the electronic supplementary material.

ESM 1 (101 KB DOCX)

## Data Availability

The data that support the findings of this study are available upon reasonable request from the corresponding author and the Netherlands Cancer Registry.
