# Supplementary material for: Longitudinal health-related quality of life profiles in esophageal cancer: insights from a nationwide prospective observational cohort study
Source: J Cancer Surviv. 2026 Jan 19;20(Suppl 2):1413–23. doi: 10.1007/s11764-026-01968-9 (PMC13744495; doi:10.1007/s11764-026-01968-9)
Supplement: Supplementary file 1 [file 11764_2026_1968_MOESM1_ESM.docx]

**Title**: Longitudinal health-related quality of life profiles in esophageal cancer: insights from a nationwide prospective observational cohort study

**Journal name**: Journal of Cancer Survivorship

**Authors**: Esther Deuning-Smit^1^*, Julien A.M. Vos^2,3^*, Ehsan Motazedi^4,5^, Ellen Engelhardt^6^, Shosha H.M. Peersman^7^, Cecile V. Baartman^8^, Annemarie M.J. Braamse^3,9^, Monique Borremans-Simons^10^, Merlijn Hutteman^11^, Ewout A. Kouwenhoven^12^, Sjoerd M. Lagarde^13^, Grard A.P. Nieuwenhuijzen^14^, Jelle P. Ruurda^15^, Marije Slingerland^16^, Fabiola Müller^3,9^, Hanneke van Laarhoven^3,8^, Pieter Coenen^4,5^.

* Authors contributed equally

**Affiliations**: 1 Department of IQ Health, Radboud University Medical Center, Nijmegen, the Netherlands; 2 Department of General Practice, Amsterdam UMC location University of Amsterdam, Amsterdam, the Netherlands; 3 Cancer Center Amsterdam, Cancer Treatment and Quality of Life, Amsterdam, the Netherlands; 4 Department of Public and Occupational Health, Amsterdam UMC location Vrije Universiteit, Amsterdam, the Netherlands; 5 Societal Participation and Health, Amsterdam Public Health Research Institute, Amsterdam, the Netherlands; 6 Division of Psychosocial Research and Epidemiology, Netherlands Cancer Institute, Amsterdam, the Netherlands; 7 Princess Máxima Center for Pediatric Oncology, Utrecht, the Netherlands; 8 Department of Medical Oncology, Amsterdam UMC location University of Amsterdam, Amsterdam, the Netherlands; 9 Department of Medical Psychology, Amsterdam UMC location University of Amsterdam, Amsterdam, the Netherlands; 10 Department of Surgery, Elisabeth-TweeSteden Hospital, Tilburg, the Netherlands; 11 Department of Surgery, Radboud University Medical Center, Nijmegen, the Netherlands; 12 Department of Surgery, ZGT Hospital, Almelo, the Netherlands; 13 Department of Surgery, Erasmus Medical Center, Rotterdam, the Netherlands; 14 Department of Surgery, Catharina Hospital, Eindhoven, the Netherlands; 15 Department of Surgery, University Medical Center Utrecht, Utrecht University, Utrecht, the Netherlands; 16 Department of Medical Oncology, Leiden University Medical Center, Leiden, the Netherlands

**Corresponding author**: Julien A.M. Vos. Post-box 22660, 1100 DD Amsterdam, the Netherlands. Tel. +31 20 566 9111. E-mail address: [j.a.m.vos@amsterdamumc.nl](mailto:j.a.m.vos@amsterdamumc.nl)

STROBE Statement—Checklist of items that should be included in reports of ***cohort studies***

|  | Item No | Recommendation | Page No |
| --- | --- | --- | --- |
| **Title and abstract** | 1 | (*a*) Indicate the study’s design with a commonly used term in the title or the abstract | 1-2 |
|  |  | (*b*) Provide in the abstract an informative and balanced summary of what was done and what was found |  |
| Introduction | | | |
| Background/rationale | 2 | Explain the scientific background and rationale for the investigation being reported | 3 |
| Objectives | 3 | State specific objectives, including any prespecified hypotheses | 3 |
| Methods | | | |
| Study design | 4 | Present key elements of study design early in the paper | 3-4 |
| Setting | 5 | Describe the setting, locations, and relevant dates, including periods of recruitment, exposure, follow-up, and data collection | 3-4 |
| Participants | 6 | (*a*) Give the eligibility criteria, and the sources and methods of selection of participants. Describe methods of follow-up | 4 |
|  |  | (*b*) For matched studies, give matching criteria and number of exposed and unexposed |  |
| Variables | 7 | Clearly define all outcomes, exposures, predictors, potential confounders, and effect modifiers. Give diagnostic criteria, if applicable | 4-5 |
| Data sources/ measurement | 8* | For each variable of interest, give sources of data and details of methods of assessment (measurement). Describe comparability of assessment methods if there is more than one group | 4-5 |
| Bias | 9 | Describe any efforts to address potential sources of bias | 4 |
| Study size | 10 | Explain how the study size was arrived at | Figure 1 |
| Quantitative variables | 11 | Explain how quantitative variables were handled in the analyses. If applicable, describe which groupings were chosen and why | 6, Supplement (p. 4) |
| Statistical methods | 12 | (*a*) Describe all statistical methods, including those used to control for confounding | 6, Supplement (p. 4) |
|  |  | (*b*) Describe any methods used to examine subgroups and interactions |  |
|  |  | (*c*) Explain how missing data were addressed |  |
|  |  | (*d*) If applicable, explain how loss to follow-up was addressed |  |
|  |  | (*e*) Describe any sensitivity analyses |  |
| Results | | |  |
| Participants | 13* | (a) Report numbers of individuals at each stage of study—eg numbers potentially eligible, examined for eligibility, confirmed eligible, included in the study, completing follow-up, and analysed | 6, Figure 1 |
|  |  | (b) Give reasons for non-participation at each stage |  |
|  |  | (c) Consider use of a flow diagram |  |
| Descriptive data | 14* | (a) Give characteristics of study participants (eg demographic, clinical, social) and information on exposures and potential confounders | 6-7, Table 1 |
|  |  | (b) Indicate number of participants with missing data for each variable of interest |  |
|  |  | (c) Summarise follow-up time (eg, average and total amount) |  |
| Outcome data | 15* | Report numbers of outcome events or summary measures over time | 7-8, Table 2, Supplement |
| Main results | 16 | (*a*) Give unadjusted estimates and, if applicable, confounder-adjusted estimates and their precision (eg, 95% confidence interval). Make clear which confounders were adjusted for and why they were included | 7-8, Figure 2, Supplement |
|  |  | (*b*) Report category boundaries when continuous variables were categorized | NA |
|  |  | (*c*) If relevant, consider translating estimates of relative risk into absolute risk for a meaningful time period | NA |
| Other analyses | 17 | Report other analyses done—eg analyses of subgroups and interactions, and sensitivity analyses | NA |
| Discussion |  |  |  |
| Key results | 18 | Summarise key results with reference to study objectives | 9 |
| Limitations | 19 | Discuss limitations of the study, taking into account sources of potential bias or imprecision. Discuss both direction and magnitude of any potential bias | 10-11 |
| Interpretation | 20 | Give a cautious overall interpretation of results considering objectives, limitations, multiplicity of analyses, results from similar studies, and other relevant evidence | 9-10 |
| Generalisability | 21 | Discuss the generalisability (external validity) of the study results | 10-11 |
| Other information |  |  |  |
| Funding | 22 | Give the source of funding and the role of the funders for the present study and, if applicable, for the original study on which the present article is based | 18 |

*Give information separately for exposed and unexposed groups.

**Data-analysis**

*Cross-sectional analyses*: We identified the optimal number of HRQoL profiles at each time point using LPA. We considered a finite Gaussian mixture model of the cross-sectional variables, using the R package ‘mclust’ [1], and explored the goodness-of-fit of the grouping with 1-5 latent profiles allowing various shapes, volumes and variance structures for the profiles. At each time point, a complete-case analysis was performed using only variables whose missing rate was no more than 40%. In case the grouping algorithm did not converge, a variable was omitted by chance from the pair of variables with the highest correlation, and this process was repeated until convergence was achieved. Considering various numbers of profiles at each time point, we thus chose the number that yielded the lowest Bayesian Information Criterion (BIC) [1, 2]. The LPA results were then used in the subsequent LTA [3]. The HRQoL values associated with the cross-sectional profiles were compared to population norms as a reference [4-6].

*Longitudinal analyses*: We performed LTA to assess how individuals grouped at each time point, while probabilistic transition was possible between the profiles at successive time points. We used the full information maximum-likelihood (FIML) approach with robust standard error estimation in Mplus [3], to obtain profile membership and transition probabilities at each time point. We used the software reported model entropy to assess the goodness-of-fit of our profiles, and considered the model fit satisfactory if its entropy was greater than 0.8 [7]. We examined the patterns of observed variable values within the identified longitudinal profiles and compared these patterns to interpret their characteristics and differences.

*Associations between the identified longitudinal profiles and demographic and clinical characteristics*

We investigated associations between the longitudinal profiles and the baseline demographic and clinical characteristics using inverse probability weighted multinomial regression analysis. Odds ratios (OR) with 95% confidence intervals (95%CI) were estimated.

Table 1 Model fits statistics (i.e., Bayesian Information Criterion (BIC)) of the latent profile analyses (LPA) for respectively 1, 2, 3, 4 and 5 latent profiles at each time point (i.e. baseline, 6-month and 12-month follow-up).

| **Number of latent profiles** | **BIC at baseline** | **BIC at 6-month** | **BIC at 12-month** |
| --- | --- | --- | --- |
| 1 | -109965.920 | -99185.590 | -85968.766 |
| 2 | -107530.879 | -97201.786 | -84048.849 |
| 3 | -106629.250 | -96290.275 | -83500.380 |
| 4 | -103867.498 | -94234.372 | -81404.371 |
| 5 | -105030.250 | -94698.125 | -82222.393 |

Table 2 Latent profiles at baseline compared to population norms

|  | **Reference** | | | **Low performing profile** | | | | **High performing profile** | | |  |
| --- | --- | --- | --- | --- | --- | --- | --- | --- | --- | --- | --- |
|  | *Mean* | *SD* | | *Mean* | | *SD* | | *Mean* | | *SD* |  |
| **EORTC QLQ-C30 subscales [4]** |  | |  | |  | |  | |  | |  |
| **Physical functioning scale** | 13.0 | 16.7 | | 29.5 | | 19.8 | | 7.9 | | 9.8 |  |
| **Role functioning scale** | 13.3 | 22.6 | | 49.1 | | 26.7 | | 9.4 | | 15.9 |  |
| **Emotional functioning scale** | 12.7 | 17.0 | | 42.1 | | 24.0 | | 15.6 | | 14.0 |  |
| **Cognitive functioning scale** | 8.7 | 13.4 | | 27.7 | | 22.9 | | 5.5 | | 10.9 |  |
| **Social functioning scale** | 7.8 | 16.8 | | 40.9 | | 25.0 | | 7.5 | | 13.0 |  |
| **Fatigue symptom scale** | 20.1 | 22.8 | | 52.4 | | 20.0 | | 17.8 | | 16.5 |  |
| **Nausea and vomiting symptom scale** | 3.5 | 9.7 | | 29.1 | | 25.4 | | 6.4 | | 12.7 |  |
| **Pain symptom scale** | 20.7 | 24.7 | | 33.7 | | 25.8 | | 10.1 | | 14.1 |  |
| **EORTC QLQ-OG25 subscales [5]** |  | |  | |  | |  | |  | |  |
| **Dysphagia scale** | 0.6 | 5.1 | | 40.6 | | 28.0 | | 17.9 | | 19.6 |  |
| **Eating scale** | 2.5 | 9.0 | | 56.4 | | 27.4 | | 24.2 | | 23.1 |  |
| **Reflux scale** | 7.0 | 15.8 | | 17.9 | | 26.0 | | 4.2 | | 10.8 |  |
| **Odynophagia scale** | 1.6 | 8.6 | | 42.0 | | 28.2 | | 20.4 | | 20.9 |  |
| **Pain and discomfort scale** | 7.8 | 15.7 | | 32.1 | | 29.7 | | 11.2 | | 16.9 |  |
| **Anxiety scale*** | - | - | | 64.6 | | 25.6 | | 44.4 | | 23.5 |  |
| **Eating with others item** | 1.4 | 8.3 | | 35.1 | | 37.1 | | 10.2 | | 21.7 |  |
| **Dry mouth item** | 11.6 | 22.7 | | 31.9 | | 30.5 | | 10.8 | | 19.5 |  |
| **Trouble with taste item** | 2.2 | 11.1 | | 39.3 | | 34.7 | | 6.8 | | 17.1 |  |
| **Body image item*** | - | - | | 26.3 | | 28.3 | | 3.1 | | 11.0 |  |
| **Trouble swallowing saliva item** | 1.0 | 7.0 | | 29.5 | | 32.2 | | 7.3 | | 16.9 |  |
| **Choked when swallowing item** | 3.2 | 12.5 | | 16.5 | | 26.1 | | 4.6 | | 13.0 |  |
| **Trouble with coughing item** | 13.2 | 23.2 | | 33.7 | | 26.8 | | 18.9 | | 21.3 |  |
| **Trouble talking item** | 1.7 | 9.0 | | 11.9 | | 18.1 | | 2.1 | | 8.5 |  |
| **Weight loss item** | 1.3 | 8.5 | | 31.9 | | 33.7 | | 11.6 | | 19.4 |  |
| **HADS subscales [8]** |  | |  | |  | |  | |  | |  |
| **Anxiety subscale** | 4.9 | 3.4 | | 8.0 | | 4.3 | | 3.7 | | 2.9 |  |
| **Depression subscale** | 5.5 | 3.9 | | 7.8 | | 3.6 | | 2.4 | | 2.5 |  |
| Note: Values represent: mean and SD. To improve interpretability, higher scores denote greater burden of complaints on quality of life or poorer performance on the functioning scales. * The authors did not administer these items since they are specific to cancer treatment and do not apply to the general population. | | | | | | | | | | |  |

Table 3 Distribution of scores on quality of life outcomes by longitudinal profiles (mean (SD))

|  | **Reference** | | **Stable high performing** | | | | | | **Stable low performing** | | | | | | **Improving** | | | | | |
| --- | --- | --- | --- | --- | --- | --- | --- | --- | --- | --- | --- | --- | --- | --- | --- | --- | --- | --- | --- | --- |
|  |  |  | ***Baseline*** | | ***6 months*** | | ***12 months*** | | ***Baseline*** | | ***6 months*** | | ***12 months*** | | ***Baseline*** | | ***6 months*** | | ***12 months*** | |
|  | *Mean* | *SD* | *Mean* | *SD* | *Mean* | *SD* | *Mean* | *SD* | *Mean* | *SD* | *Mean* | *SD* | *Mean* | *SD* | *Mean* | *SD* | *Mean* | *SD* | *Mean* | *SD* |
| **EORTC-QLQ-C30 subscales[4]** |  |  |  |  |  |  |  |  |  |  |  |  |  |  |  |  |  |  |  |  |
| **Physical functioning scale** | 13.0 | 16.7 | 7.9 | 10.0 | 14.8 | 13.6 | 10.9 | 11.4 | 33.8 | 21.3 | 39.8 | 23.9 | 41.0 | 21.8 | 27.5 | 18.5 | 19.2 | 13.0 | 14.5 | 13.0 |
| **Role functioning scale** | 13.3 | 22.6 | 8.6 | 15.5 | 18.4 | 21.7 | 11.9 | 18.3 | 54.4 | 26.6 | 57.1 | 28.8 | 48.7 | 21.6 | 48.0 | 26.1 | 30.1 | 24.1 | 17.2 | 22.6 |
| **Emotional functioning scale** | 12.7 | 17.0 | 14.4 | 13.8 | 9.1 | 12.2 | 8.3 | 11.9 | 51.4 | 28.1 | 40.2 | 22.2 | 33.1 | 21.6 | 37.4 | 20.6 | 21.0 | 13.9 | 15.6 | 15.6 |
| **Cognitive functioning scale** | 8.7 | 13.4 | 4.8 | 10.3 | 6.8 | 11.7 | 7.8 | 12.3 | 37.2 | 23.8 | 36.2 | 24.8 | 32.7 | 21.8 | 24.9 | 21.3 | 12.4 | 14.3 | 12.9 | 16.5 |
| **Social functioning scale** | 7.8 | 16.8 | 7.1 | 13.0 | 11.9 | 15.1 | 6.6 | 12.4 | 43.9 | 25.3 | 52.9 | 26.8 | 45.5 | 16.7 | 41.2 | 24.4 | 19.9 | 23.3 | 8.6 | 14.2 |
| **Fatigue symptom scale** | 20.1 | 22.8 | 17.4 | 16.4 | 23.0 | 17.4 | 19.0 | 15.6 | 54.8 | 17.2 | 56.0 | 22.7 | 57.7 | 21.3 | 52.0 | 21.9 | 30.5 | 15.4 | 26.2 | 12.4 |
| **Nausea and vomiting symptom scale** | 3.5 | 9.7 | 5.9 | 11.7 | 8.1 | 14.2 | 6.1 | 12.1 | 28.3 | 25.9 | 24.4 | 26.2 | 20.5 | 19.0 | 29.9 | 25.5 | 11.3 | 14.5 | 3.8 | 7.1 |
| **Pain symptom scale** | 20.7 | 24.7 | 9.3 | 13.8 | 10.9 | 16.2 | 7.9 | 13.6 | 40.6 | 27.2 | 39.7 | 21.6 | 39.7 | 25.9 | 31.1 | 24.7 | 14.5 | 17.1 | 11.3 | 16.3 |
| **EORTC-QLQ-OG25 subscales [5]** |  |  |  |  |  |  |  |  |  |  |  |  |  |  |  |  |  |  |  |  |
| **Dysphagia scale** | 0.6 | 5.1 | 17.4 | 20.1 | 9.3 | 14.5 | 5.7 | 9.0 | 38.9 | 27.2 | 46.4 | 30.1 | 27.4 | 23.1 | 42.5 | 28.6 | 10.8 | 15.0 | 8.2 | 9.1 |
| **Eating scale** | 2.5 | 9.0 | 23.8 | 24.0 | 20.5 | 21.3 | 14.2 | 15.7 | 53.1 | 29.8 | 60.1 | 24.4 | 49.7 | 22.0 | 58.3 | 26.9 | 24.2 | 18.7 | 18.5 | 16.1 |
| **Reflux scale** | 7.0 | 15.8 | 4.0 | 10.4 | 7.2 | 14.7 | 7.3 | 13.5 | 26.7 | 29.6 | 21.3 | 31.5 | 21.8 | 25.7 | 13.6 | 23.7 | 8.6 | 14.8 | 14.5 | 21.8 |
| **Odynophagia scale** | 1.6 | 8.6 | 19.5 | 21.1 | 8.0 | 12.6 | 4.7 | 9.4 | 42.8 | 29.6 | 39.9 | 30.9 | 30.8 | 23.4 | 41.5 | 28.3 | 7.5 | 12.0 | 9.7 | 11.2 |
| **Pain and discomfort scale** | 7.8 | 15.7 | 10.0 | 15.5 | 7.4 | 14.0 | 8.5 | 14.8 | 34.4 | 30.6 | 30.5 | 26.8 | 32.1 | 20.5 | 31.4 | 30.0 | 8.6 | 16.6 | 9.1 | 16.0 |
| **Anxiety scale*** | - | - | 42.6 | 23.6 | 24.0 | 20.6 | 18.1 | 19.3 | 70.0 | 25.7 | 60.3 | 22.0 | 48.1 | 25.5 | 63.0 | 24.6 | 37.1 | 20.5 | 27.4 | 21.8 |
| **Eating with others item** | 1.4 | 8.3 | 10.0 | 21.2 | 6.8 | 16.2 | 4.9 | 13.5 | 34.4 | 38.6 | 40.5 | 37.8 | 40.0 | 39.7 | 35.7 | 37.0 | 12.9 | 25.4 | 9.7 | 17.6 |
| **Dry mouth item** | 11.6 | 22.7 | 10.8 | 19.7 | 12.1 | 20.6 | 11.3 | 19.3 | 35.6 | 26.2 | 51.7 | 30.3 | 38.5 | 29.4 | 31.6 | 32.7 | 12.9 | 18.6 | 10.8 | 18.0 |
| **Trouble with taste item** | 2.2 | 11.1 | 7.1 | 17.4 | 12.0 | 21.6 | 6.8 | 15.0 | 33.3 | 35.0 | 40.5 | 27.8 | 39.7 | 35.3 | 41.2 | 35.7 | 9.7 | 19.6 | 9.7 | 21.4 |
| **Body image item*** | - | - | 3.2 | 11.4 | 6.0 | 14.0 | 4.0 | 11.6 | 18.9 | 22.6 | 35.6 | 36.7 | 34.6 | 30.5 | 31.6 | 30.6 | 10.8 | 20.0 | 8.6 | 17.1 |
| **Trouble swallowing saliva item** | 1.0 | 7.0 | 6.9 | 16.5 | 5.4 | 14.8 | 2.5 | 8.7 | 33.3 | 35.0 | 32.1 | 30.7 | 24.4 | 30.6 | 27.7 | 31.0 | 9.7 | 19.6 | 4.3 | 11.4 |
| **Choked when swallowing item** | 3.2 | 12.5 | 4.7 | 13.4 | 5.5 | 13.8 | 5.6 | 12.8 | 21.1 | 28.3 | 27.4 | 32.8 | 23.1 | 26.3 | 15.8 | 25.8 | 14.4 | 18.9 | 9.7 | 17.6 |
| **Trouble with coughing item** | 13.2 | 23.2 | 19.1 | 21.8 | 28.0 | 29.1 | 20.4 | 22.7 | 37.8 | 32.4 | 59.3 | 37.4 | 48.7 | 31.6 | 31.6 | 23.5 | 35.5 | 25.7 | 26.9 | 23.4 |
| **Trouble talking item** | 1.7 | 9.0 | 2.2 | 8.3 | 6.2 | 17.2 | 3.9 | 11.9 | 17.8 | 19.0 | 23.8 | 31.2 | 23.1 | 35.0 | 9.6 | 17.6 | 9.7 | 17.6 | 5.4 | 12.5 |
| **Weight loss item** | 1.3 | 8.5 | 10.9 | 19.2 | 11.9 | 19.0 | 9.5 | 15.6 | 28.9 | 25.9 | 41.7 | 35.9 | 34.6 | 31.9 | 34.5 | 37.6 | 24.7 | 29.8 | 12.9 | 18.6 |
| **HADS subscales [8]** |  |  |  |  |  |  |  |  |  |  |  |  |  |  |  |  |  |  |  |  |
| **Anxiety subscale** | 4.9 | 3.4 | 3.5 | 2.9 | 2.5 | 2.6 | 2.3 | 2.7 | 9.8 | 4.3 | 8.4 | 3.3 | 7.2 | 3.8 | 7.0 | 4.0 | 4.8 | 2.8 | 4.0 | 3.4 |
| **Depression subscale** | 5.5 | 3.9 | 2.2 | 2.5 | 2.5 | 2.4 | 2.3 | 2.5 | 9.2 | 4.1 | 9.3 | 3.9 | 8.5 | 3.6 | 7.2 | 3.1 | 3.9 | 2.8 | 3.4 | 2.4 |

Table 3 Distribution of scores on quality of life outcomes by longitudinal profiles (mean (SD))

|  | **Deteriorating** | | | | | | **Fluctuating** | | | | | |
| --- | --- | --- | --- | --- | --- | --- | --- | --- | --- | --- | --- | --- |
|  | ***Baseline*** | | ***6 months*** | | ***12 months*** | | ***Baseline*** | | ***6 months*** | | ***12 months*** | |
|  | *Mean* | *SD* | *Mean* | *SD* | *Mean* | *SD* | *Mean* | *SD* | *Mean* | *SD* | *Mean* | *SD* |
| **EORTC QLQ-C30 subscales [4]** |  |  |  |  |  |  |  |  |  |  |  |  |
| **Physical functioning scale** | 8.4 | 8.9 | 34.6 | 23.4 | 33.7 | 20.0 | 10.3 | 14.9 | 34.8 | 20.2 | 18.2 | 15.9 |
| **Role functioning scale** | 15.2 | 17.9 | 49.3 | 33.8 | 49.7 | 27.1 | 12.9 | 20.5 | 54.8 | 27.1 | 24.6 | 25.6 |
| **Emotional functioning scale** | 20.2 | 13.9 | 32.7 | 23.0 | 36.5 | 19.4 | 25.3 | 17.4 | 25.7 | 21.3 | 12.1 | 17.7 |
| **Cognitive functioning scale** | 9.9 | 12.9 | 25.3 | 22.5 | 31.4 | 24.0 | 6.5 | 11.9 | 14.9 | 15.9 | 11.4 | 16.0 |
| **Social functioning scale** | 9.9 | 12.5 | 37.3 | 26.1 | 38.7 | 25.6 | 11.3 | 17.4 | 35.5 | 26.3 | 13.2 | 19.8 |
| **Fatigue symptom scale** | 20.6 | 16.3 | 50.7 | 23.4 | 54.3 | 18.2 | 22.9 | 20.5 | 52.9 | 18.7 | 29.8 | 20.7 |
| **Nausea and vomiting symptom scale** | 7.3 | 14.4 | 25.7 | 27.5 | 29.4 | 25.4 | 14.0 | 21.1 | 20.2 | 17.8 | 9.6 | 14.8 |
| **Pain symptom scale** | 15.2 | 15.2 | 32.9 | 27.8 | 33.6 | 27.2 | 12.9 | 17.6 | 31.1 | 27.2 | 16.7 | 26.6 |
| **EORTC QLQ-OG25 subscales [5]** |  |  |  |  |  |  |  |  |  |  |  |  |
| **Dysphagia scale** | 19.1 | 15.8 | 28.8 | 26.5 | 26.2 | 27.0 | 23.1 | 22.1 | 27.6 | 24.0 | 9.8 | 12.7 |
| **Eating scale** | 25.5 | 17.6 | 47.8 | 29.4 | 48.7 | 25.1 | 31.5 | 25.2 | 50.0 | 21.4 | 29.5 | 22.1 |
| **Reflux scale** | 4.7 | 13.6 | 13.2 | 19.6 | 24.1 | 28.1 | 7.0 | 13.5 | 16.2 | 21.6 | 12.2 | 19.1 |
| **Odynophagia scale** | 24.4 | 18.0 | 22.5 | 22.9 | 27.2 | 21.5 | 27.4 | 24.6 | 19.0 | 18.3 | 10.8 | 15.8 |
| **Pain and discomfort scale** | 15.2 | 20.0 | 30.3 | 26.0 | 33.9 | 29.4 | 20.4 | 23.8 | 13.5 | 18.8 | 17.6 | 20.4 |
| **Anxiety scale** | 50.9 | 22.2 | 50.7 | 26.5 | 58.3 | 26.0 | 53.8 | 22.7 | 41.4 | 23.4 | 24.8 | 16.0 |
| **Eating with others item** | 8.8 | 21.4 | 21.6 | 32.4 | 28.7 | 35.3 | 19.4 | 30.8 | 22.5 | 30.5 | 15.3 | 21.7 |
| **Dry mouth item** | 10.5 | 18.0 | 41.4 | 31.1 | 36.8 | 29.1 | 11.8 | 22.0 | 44.1 | 36.1 | 25.2 | 30.8 |
| **Trouble with taste item** | 7.0 | 17.5 | 34.7 | 34.4 | 37.4 | 30.9 | 11.8 | 22.0 | 31.4 | 31.3 | 19.8 | 24.2 |
| **Body image item** | 2.9 | 9.5 | 28.4 | 31.1 | 34.5 | 31.2 | 4.3 | 11.4 | 19.8 | 24.2 | 15.3 | 23.0 |
| **Trouble swallowing saliva item** | 10.5 | 20.1 | 23.6 | 28.4 | 23.6 | 27.7 | 9.7 | 19.6 | 14.4 | 24.3 | 9.0 | 16.9 |
| **Choked when swallowing item** | 5.3 | 12.3 | 18.0 | 22.9 | 20.8 | 26.6 | 2.2 | 8.3 | 12.6 | 18.2 | 9.9 | 17.3 |
| **Trouble with coughing item** | 19.6 | 18.8 | 47.6 | 27.5 | 42.9 | 31.0 | 18.3 | 22.5 | 47.4 | 27.5 | 26.1 | 21.0 |
| **Trouble talking item** | 2.4 | 10.7 | 22.2 | 29.2 | 19.0 | 26.9 | 1.1 | 6.0 | 14.0 | 25.3 | 7.2 | 16.0 |
| **Weight loss item** | 17.5 | 21.0 | 31.1 | 30.0 | 42.7 | 35.5 | 10.8 | 20.0 | 23.7 | 25.6 | 11.7 | 19.6 |
| **HADS subscales [8]** |  |  |  |  |  |  |  |  |  |  |  |  |
| **Anxiety subscale** | 4.4 | 2.8 | 6.3 | 3.8 | 6.8 | 3.4 | 5.5 | 3.5 | 5.1 | 3.6 | 3.4 | 2.9 |
| **Depression subscale** | 3.0 | 2.5 | 6.8 | 3.9 | 8.0 | 3.7 | 3.5 | 3.5 | 6.1 | 3.5 | 3.4 | 2.6 |
| Note: To improve interpretability, higher scores denote greater burden of complaints on quality of life or poorer performance on the functioning scales. * The authors did not administer these items since they are specific to cancer treatment and do not apply to the general population. | | | | | | | | | | | | |

Table 4 Descriptive statistics of all longitudinal profiles

|  |  | **Stable high** | **Stable low** | **Improving** | **Deteriorating** | **Fluctuating** |
| --- | --- | --- | --- | --- | --- | --- |
| Total |  | 395 (65%) | 30 (5%) | 59 (10%) | 83 (14%) | 38 (6%) |
|  |  |  |  |  |  |  |
| Sex | Males | 332 (84%) | 20 (67%) | 44 (75%) | 70 (84%) | 31 (82%) |
|  | Female | 63 (16%) | 10 (33%) | 15 (25%) | 13 (16%) | 7 (18%) |
|  |  |  |  |  |  |  |
| Education level | Low | 150 (44%) | 15 (50%) | 27 (46%) | 23 (40%) | 15 (48%) |
|  | Middle | 111 (32%) | 9 (30%) | 22 (37%) | 22 (39%) | 8 (26%) |
|  | High | 82 (24%) | 6 (20%) | 10 (17%) | 12 (21%) | 8 (26%) |
|  |  |  |  |  |  |  |
| Marital status | Married / living together | 281 (81%) | 22 (73%) | 49 (83%) | 44 (76%) | 23 (77%) |
|  | Single | 64 (19%) | 8 (27%) | 10 (17%) | 14 (24%) | 7 (23%) |
|  |  |  |  |  |  |  |
| Tumor stage | I/II | 225 (57%) | 17 (57%) | 30 (51%) | 48 (58%) | 17 (45%) |
|  | III | 170 (43%) | 13 (43%) | 29 (49%) | 35 (42%) | 21 (55%) |
|  |  |  |  |  |  |  |
| Topography | Upper/middle | 50 (13%) | 4 (13%) | 7 (12%) | 12 (14%) | 3 (8%) |
|  | Lower | 300 (76%) | 23 (77%) | 48 (81%) | 62 (75%) | 32 (84%) |
|  | Other | 45 (11%) | 3 (10%) | 4 (7%) | 9 (11%) | 3 (8%) |
|  |  |  |  |  |  |  |
| Surgery | No | 114 (29%) | 6 (20%) | 21 (37%) | 18 (22%) | 5 (13%) |
|  | Yes | 281 (71%) | 24 (80%) | 38 (64%) | 65 (78%) | 33 (87%) |
|  |  |  |  |  |  |  |
| Age |  | 66.8 (7.8) | 64.5 (9.0) | 66.2 (8.7) | 67.1 (8.5) | 66.0 (6.8) |
| BMI |  | 26,8 (4.2) | 26.6 (4.5) | 26.1 (5.4) | 25.8 (3.6) | 26.4 (4.3) |
| No. surgical complications |  | 1.7 (2.3) | 1.4 (2.3) | 1.4 (1.9) | 1.9 (2.3) | 1.0 (2.0) |
| Note. Mean and standard deviation for continuous variables and number of participants and percentages for categorical variables. | | | | | | |

Table 5 Multinominal univariate analysis showing the odds of being in any of the four longitudinal profiles.

|  |  | **Stable low** | **Improving** | **Deteriorating** | **Fluctuating** |
| --- | --- | --- | --- | --- | --- |
| Sex | Males | Reference | Reference | Reference | Reference |
|  | Female | **2.63 [1.18; 5.89]** | 1.80 [0.94; 3.42] | 0.98 [0.51; 1.88] | 1.19 [0.50; 2.82] |
|  |  |  |  |  |  |
| Education level | Low | Reference | Reference | Reference | Reference |
|  | Middle | 0.81 [0.34; 1.92] | 1.10 [0.56; 2.04] | 1.29 [0.69; 2.44] | 0.72 [0.30; 1.76] |
|  | High | 0.73 [0.27; 1.96] | 0.67 [0.31; 1.47] | 0.95 [0.45; 2.02] | 0.98 [0.40; 2.40] |
|  |  |  |  |  |  |
| Marital status | Married / living together | Reference | Reference | Reference | Reference |
|  | Single | 1.60 [0.68; 3.75] | 0.89 [0.43; 1.86] | 1.40 [0.72; 1.70] | 1.34 [0.55; 3.25] |
|  |  |  |  |  |  |
| Tumor stadium | I/II | Reference | Reference | Reference | Reference |
|  | III | 1.01 [0.48; 2.14] | 1.28 [0.74; 2.21] | 0.97 [0.60; 1.56] | 1.64 [0.84;3.19] |
|  |  |  |  |  |  |
| Tumor topography | Upper/middle | Reference | Reference | Reference | Reference |
|  | Lower | 0.96 [0.32; 2.89] | 1.14 [0.49; 2.67] | 0.86 [0.43; 1.71] | 1.78 [0.52; 6.03] |
|  | Other | 0.83 [0.18; 3.93] | 0.64 [0.17; 2.31] | 0.83 [0.32; 2.16] | 1.11 [0.21; 5.79] |
|  |  |  |  |  |  |
| Surgery* | No | Reference | Reference | Reference | Reference |
|  | Yes | 1.62 [0.65; 4.08] | 0.73 [0.41; 1.31] | 1.47 [0.83; 2.58] | **2.68 [1.02; 7.03]** |
|  |  |  |  |  |  |
| Age |  | 0.97 [0.92; 1.01] | 0.99 [0.96; 1.03] | 1.00 [0.97; 1.03] | 0.99 [0.95; 1.03] |
|  |  |  |  |  |  |
| BMI |  | 0.99 [0.90; 1.09] | 0.96 [0.90; 1.03] | **0.91 [0.85; 0.98]** | 0.95 [0.87; 1.04] |
|  |  |  |  |  |  |
| No. surgical complications |  | 0.93 [0.75; 1.15] | 0.93 [0.78; 1.11] | 1.03 [0.91; 1.18] | 0.82 [0.64; 1.06] |
| Note. Odds ratios (OR) with 95% confidence intervals (95% CI) are shown (with stable high as reference category). Statistically significant findings (p<0.05) are printed in bold. * The no-surgery group was chosen as the reference to facilitate the interpretation of ORs. | | | | | |

**References**

1.	Scrucca L, Fop M, Murphy TB, Raftery. AE. mclust 5: Clustering, classification and density estimation using Gaussian finite mixture models. RJ. 2016;8(1):289-317.

2.	C F, A.E R. Model-Based Clustering, Discriminant Analysis, and Density Estimation. Journal of the American Statistical Association. 2002;97:611-31.

3.	Muthén B, Asparouhov T. Latent transition analysis with random intercepts (RI-LTA). Psychol Methods. 2022 27(1):1-16.

4.	de Ligt KM, Aaronson NK, Liegl G, Nolte S. Updated normative data for the EORTC QLQ-C30 in the general Dutch population by age and sex: a cross-sectional panel research study. Qual Life Res. 2023;32(9):2477-87.

5.	van der Schaaf M, Derogar M, Lagergren P. Reference values of oesophago-gastric symptoms (EORTC QLQ-OG25) in a population-based setting. Eur J Cancer. 2012;48(11):1602-7.

6.	Hinz A, Brähler E. Normative values for the hospital anxiety and depression scale (HADS) in the general German population. J Psychosom Res. 2011;71(2):74-8.

7.	Sinha P, Furfaro D, Cummings MJ, Abrams D, Delucchi K, Maddali MV, et al. Latent class analysis reveals COVID-19-related acute respiratory distress syndrome subgroups with differential responses to corticosteroids. American journal of respiratory and critical care medicine. 2021;204(11):1274-85.

8.	Zigmond AS, Snaith RP. The hospital anxiety and depression scale. Acta Psychiatr Scand. 1983;67(6):361-70.
